# How sequence populations persist inside bacterial genomes

**DOI:** 10.1093/genetics/iyab027

**Published:** 2021-03-16

**Authors:** Hye Jin Park, Chaitanya S Gokhale, Frederic Bertels

**Affiliations:** 1 Department of Evolutionary Theory, Max Planck Institute for Evolutionary Biology, Plön, 24306, Germany; 2 Asia Pacific Center for Theoretical Physics, Pohang, 37673, Korea; 3 Department of Physics, POSTECH, Pohang, 37673, Korea; 4 Research Group for Theoretical Models of Eco-evolutionary Dynamics, Department of Evolutionary Theory, Max Planck Institute for Evolutionary Biology, Plön, 24306, Germany; 5 Research Group for Microbial Molecular Evolution, Department of Microbial Population Biology, Max Planck Institute for Evolutionary Biology, Plön, 24306, Germany

**Keywords:** REPINs, mobile elements, evolution, transposons

## Abstract

Compared to their eukaryotic counterparts, bacterial genomes are small and contain extremely tightly packed genes. Repetitive sequences are rare but not completely absent. One of the most common repeat families is REPINs. REPINs can replicate in the host genome and form populations that persist for millions of years. Here, we model the interactions of these intragenomic sequence populations with the bacterial host. We first confirm well-established results, in the presence and absence of horizontal gene transfer (*hgt*) sequence populations either expand until they drive the host to extinction or the sequence population gets purged from the genome. We then show that a sequence population can be stably maintained, when each individual sequence provides a benefit that decreases with increasing sequence population size. Maintaining a sequence population of stable size also requires the replication of the sequence population to be costly to the host, otherwise the sequence population size will increase indefinitely. Surprisingly, in regimes with high *hgt* rates, the benefit conferred by the sequence population does not have to exceed the damage it causes to its host. Our analyses provide a plausible scenario for the persistence of sequence populations in bacterial genomes. We also hypothesize a limited biologically relevant parameter range for the provided benefit, which can be tested in future experiments.

## Introduction

Repetitive sequences can be found in most genomes. They are particularly abundant in eukaryotes, where often only a small proportion of the genome encodes for host proteins (Jurka *et al.* 2007). In contrast, about 90% of a typical bacterial genome encodes for host proteins (Silby *et al.* 2009). The extragenic space is mostly taken up by rRNA, tRNA, transcription and translation promoters, repressors, and terminators (Rogozin *et al.* 2002). Yet, repetitive sequences can also be found in the extragenic space of many bacteria (Treangen *et al.* 2009).

Short repetitive sequences were first identified in *Escherichia coli* in the early 1980s (Higgins *et al.* 1982). Then, due to their characteristics, they were called REPs, short for **r**epetitive **e**xtragenic **p**alindromic sequences (Stern *et al.* 1984). It was unclear if REP sequences fulfill a functional role in the host bacterium and if so what kind of function this might be. Numerous studies found REP sequences to be involved in different biological processes, for example in transcription termination, RNA stabilization, gyrase, and integration host factor binding, as well as nucleoid folding (Higgins *et al.* 1982; Newbury *et al.* 1987; Yang and Ames 1988; Boccard and Prentki 1993; Espéli *et al.* 2001; Qian *et al.* 2015). However, whether the identified functions are locally co-opted, or common to all REP sequences and therefore able to explain the presence of REP sequences in the bacterial genome, is not clear.

To determine whether a function is incidental or whether it can explain the persistence and emergence of an entire sequence class requires the understanding of the evolution of REP sequences. A study in *Pseudomonas fluorescens* SBW25 showed that REP sequences are not evolutionarily relevant units (Bertels and Rainey 2011b), but a part of a larger replicative unit, called REPIN (**REP** doublet forming hairp**in**). REPINs consist of two inverted REP sequences separated by a short and highly diverse spacer region. This arrangement allows REPINs to form hairpins in single-stranded DNA or RNA. REP singlets also exist, but these are usually decaying remnants of full-length REPINs. REPINs are nonautonomous transposable elements that are duplicated by RAYT (**R**EP **a**ssociated t**y**rosine **t**ransposase) proteins (Nunvar *et al.* 2010; Bertels and Rainey 2011b; Ton-Hoang *et al.* 2012).

RAYT transposases are single-copy genes that have been vertically inherited for millions of years (Bertels *et al.* 2017a), making RAYTs domesticated transposases. Despite the domestication of the RAYT transposase by the bacterium, RAYTs have not lost their association to REPINs and actively replicate REPINs albeit at very low rates (Bertels *et al.* 2017b).

Although the RAYT transposase’s exact function is unknown, it is conceivable that formerly parasitic genes are domesticated by the host. It is much less clear how a population of replicating sequences can be maintained in a bacterial genome over long periods of time. There is a large body of literature on the persistence of transposable elements (TEs). In the 1980s research was mostly focused on how it is possible to maintain TEs in sexually reproducing eukaryotic genomes (Doolittle and Sapienza 1980; Hickey 1982; Charlesworth and Charlesworth 1983; Wright and Finnegan 2001). These studies showed that beneficial effects need not be invoked to explain the presence of TEs in the genome. Instead, if the TE copies or transposes itself from one sister chromatid to the other during meiosis, TEs can even reduce the host’s fitness by up to 50% and still spread through the host population.

TEs are much rarer in asexually reproducing prokaryotic genomes than in sexually reproducing eukaryotic genomes. Nevertheless, studies of TEs in asexually reproducing organisms followed shortly after the first studies on eukaryotes (Sawyer and Hartl 1986). The authors assume, similar to sexually reproducing organisms, “*that the TE performs no function for the host and, that the reduction in fitness with increased copy number is due to effects such as impairment of beneficial genes by transposition or homologous recombination.”* These models can explain the distribution of simple TEs such as insertion sequences (ISs), and even short repetitive sequences assumed to act as promoters (mobile promoters, MPs) as long as there is replicative horizontal gene transfer (*hgt*) (Sawyer and Hartl 1986; Dolgin and Charlesworth 2006; Matus-Garcia *et al.* 2012; Bichsel *et al.* 2013; van Passel *et al.* 2014).

As more and more sequence data became available, it was noticed that TEs often cause beneficial mutations in prokaryotic genomes (Schneider *et al.* 2000). When incorporating the mutational effect of TEs into models, analyses showed that mutation rates increased by TEs can elevate TE persistence time in bacterial genomes in novel or fluctuating environments (Martiel and Blot 2002; Edwards and Brookfield 2003; McGraw and Brookfield 2006; Startek *et al.* 2013). TEs can theoretically be maintained at intermediate numbers if the environment fluctuates regularly (Startek *et al.* 2013). However, there are numerous issues with this result. As the authors point out, TEs will not be maintained through this mechanism over long evolutionary time periods.

One reason is that nonautonomous TEs are expected to quickly evolve by inactivating mutations of the encoded transposase. Nonautonomous elements cannot produce a transposase protein, but can be the target of transposases produced by autonomous elements. The evolution of nonautonomous elements will quickly lead to the extinction of full-length elements, making the long-term survival of TEs in prokaryotic genomes unlikely, consistent with the transient nature of prokaryotic TEs in sequenced bacterial genomes (Sawyer and Hartl 1986; Startek *et al.* 2013).

A second reason is that increasing mutation rates by ISs is not a viable strategy over long evolutionary time periods. Each time a beneficial mutation is generated through the insertion of a TE, the transposition rate increases. Increasing the transposition rate will, of course, increase the mutation rate and lead to high costs for the cell. Hence, increasing mutation rates by modifying the DNA repair system should, in the long term, be a less costly route of adapting to novel environments (Wielgoss *et al.* 2013; Consuegra *et al.* 2021).

**Figure 1:**
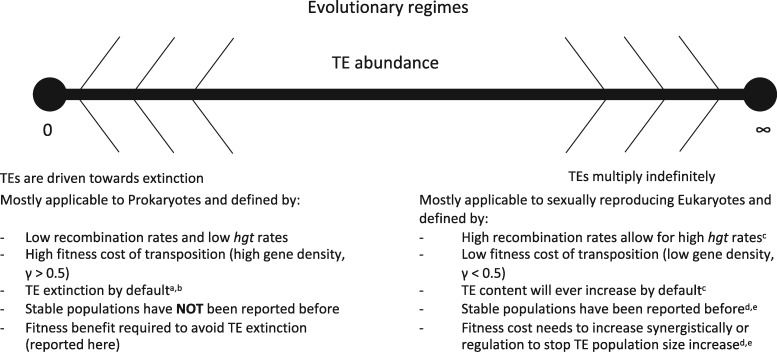
Previous research shows there are two trivial outcomes for transposable element evolution. In prokaryotes, transposable elements go extinct by default (Dolgin and Charlesworth 2006). In eukaryotes, transposable elements tend to increase indefinitely until eventually the TE population collapses and a large part of the genome is lost. The TE population size will then increase again until eventual collapse. This has been shown to have happened in birds and mammals (Kapusta *et al.* 2017). Superscripts indicate the following references: a (Sawyer and Hartl 1986), b (RANKIN *et al.* 2010), c (Hickey 1982), d (Charlesworth and Charlesworth 1983), e (Charlesworth and Langley 1989).

In eukaryotes stably maintained sequence populations exist in *Drosophila* populations (Charlesworth and Charlesworth 1983; Charlesworth and Langley 1989). A stable population can only be obtained when the accumulation of TEs is stopped (Figure 1). This can be achieved by either an exponentially increasing fitness cost of TEs or the down regulation of transposition rates. Without *hgt* or recombination a stable equilibrium of intermediate TE numbers cannot be maintained (Wright and Schoen 1999).

To obtain stable sequence populations in prokaryotes, the high cost of transposition has to be alleviated to prevent the extinction of TEs (Figure 1). Since until recently stable sequence populations in prokaryotes have not been observed, no mathematical model has been proposed to explain the persistence of intermediate numbers of TEs over long time periods.

Currently, REPINs are to our knowledge the only intragenomic sequence population that is stably maintained in prokaryotes. REPINs have been maintained in the genome for millions of years (Bertels *et al.* 2017a,b) and mean and mode of the population size is far greater than 0 in *E. coli*.

**Figure 2:**
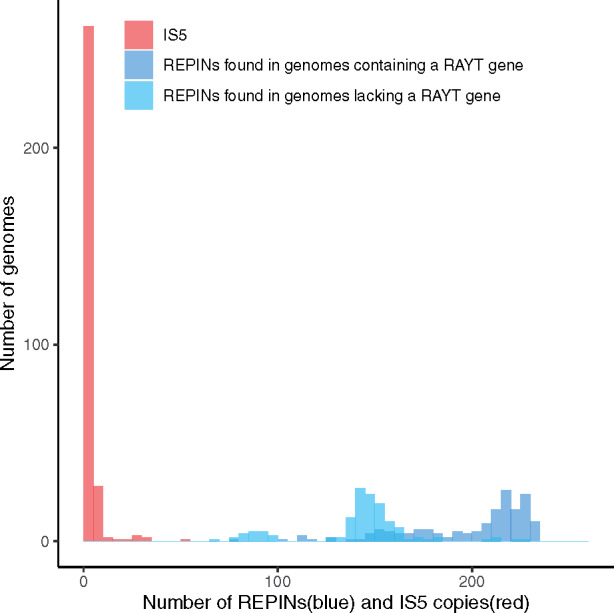
Distribution of IS*5* elements (red) compared to REPINs (blue) across 300 de-replicated *E. coli* genomes. To display the REPIN numbers, *E. coli* genomes are divided into two categories. Genomes that contain the RAYT transposase gene (dark blue) and genomes that do not (light blue). REPINs are more common in genomes that contain a RAYT gene compared to genomes that do not contain a RAYT gene. The distribution of REPIN numbers does not overlap with the distribution of IS*5* elements (*i.e.*, IS*5* occurs at most 53 times per *E. coli* genome, yet there are at least 69 REPINs present per *E. coli* genome).

For example, across 20 representative *E. coli* strains the minimum REPIN number is 96, and the average is 156 (Touchon *et al.* 2009), whereas IS*5* is only present in four of 20 strains. This pattern also holds for larger *E. coli* strain collections. In a selection of 300 *E. coli* genomes only 44% (133) contain one or more IS*5* genes (Figure 2). The maximum number of IS*5* copies is 53. In contrast, across the same strain collection, the minimum REPIN number is 69 and the maximum 235. RAYT containing [the transposase responsible for REPIN transposition (Nunvar *et al.* 2010; Bertels and Rainey 2011b; Messing *et al.* 2012)] genomes harbor more REPINs than genomes lacking the RAYT transposase responsible for REPIN dissemination. There is no overlap between the REPIN distribution and the IS*5* distribution in *E. coli*, which strongly suggests that fundamentally different evolutionary processes maintain REPINs inside bacterial genomes compared to ISs.

Our study aims to understand the conditions that allow the maintenance of intermediate REPIN numbers. We start by devising a simple model for REPIN evolution. In agreement with previous work, we show that in our model the bacterial population will either be driven to extinction by the cost of the transposition activity of an ever-increasing intragenomic sequence population or the sequence population will be lost from the bacterial population, with and without nonreplicative *hgt* (Figure 1). However, persistence of intermediate numbers is possible when each sequence provides a small benefit to the host bacterium, decreasing as the sequence number per genome increases. Interestingly, for high nonreplicative *hgt* rates, sequence populations can persist even if the caused harm outweighs the fitness benefit provided to the host. Together, our analyses provide testable hypotheses to explain the persistence of intragenomic sequence populations in bacteria.

## Materials and methods

### REPIN and IS*5* distribution in *E. coli*

We downloaded 1165 *E. coli* genomes from NCBI (https://www.ncbi.nlm.nih.gov/) on the 27th of February 2020 using the following query “*(“Escherichia coli”[Organism] OR Escherichia coli[All Fields]) AND (latest[filter] AND (all[filter] NOT” derived from surveillance project”[filter] AND all[filter] NOT anomalous[filter])) AND (”complete genome”[filter] OR” chromosome level”[filter]) AND” has annotation”[Properties]*”. We then de-replicated those genomes to make sure that all nucleotide sequences of all genomes differed by at least 0.5% (Mash distance) using the “Assembly de-replicator” (downloaded on the 27th of February 2020). A selection of 300 genomes remained. The sequences can be downloaded using the code provided at GitHub.

For all genomes, REPINs were identified by first determining the most common 21 bp long sequences in *E. coli* O15: H11 strain 90-9272 (GATGCGGCGTGAACGCCTTAT). All related sequences that differ in at most one position are identified recursively for this seed sequence until no more new sequences are found. This procedure was repeated with the same 21 bp long seed sequence for all 300 *E. coli* genomes.

IS*5* sequences were identified using TBLASTN in BLAST+ (Camacho *et al.* 2009) (version 2.10.0) with an *e*-value threshold of 1e-90 and the IS*5* protein with NCBI accession number QEF05883.1 as a query. Similarly, RAYT sequences were identified with TBLASTN using the YafM protein from *E. coli* K-12 MG1655 as a query and an *e*-value threshold of 1e-90. We chose low *e*-value thresholds to ensure that we only analyze full-length and likely functional genes that mainly evolved inside the *E. coli* species.

The analyses can be done with the RAREFAN webtool.

### Local REPIN amplification rate λ

REPINs are often found in two or more tandem repeat copies (Bachellier *et al.* 1997; Bertels and Rainey 2011b). Hence, REPINs can get locally amplified or deleted. To estimate the local amplification and deletion rates in the genome, we consulted mutation accumulation data from *E. coli* MG1655 (Foster *et al.* 2015). In this experiment, the authors started 50 parallel mutation accumulation lines from a single *E. coli* MG1655 wild-type clone (strain PFM2m). These 50 lines were grown on minimal medium and serially transferred about 220 times through single-cell bottlenecks. Between bottlenecks, the cells grew for about 28 generations. The final bacterial clones experienced about 6160 cell divisions from the start to the end of the experiment (Lee *et al.* 2012; Foster *et al.* 2015). At the end of the experiment, the authors observed 277 single base-pair substitutions across the 50 individual mutation accumulation lines and based on this data estimated a per genome mutation rate of $277(\mathrm{substitutions})/(6160(\mathrm{generations})\times 50(\mathrm{lines}))\approx 0.9\times 10^{-3}$.

Using the same logic, and further data from (Lee *et al.* 2016), we can estimate the local amplification rates of REPINs. Across the experiment, they only observed a single large indel that involved REPINs and hence is relevant for the estimation of local REPIN amplifications and deletions (*λ*). We analyzed the Illumina sequence data with breseq (Deatherage and Barrick 2014) to verify the presence of a mutation in a REPIN cluster. This event occurred in M2M-85 (SRA accession number: SRR2169198) at position 4295870.4296434 in the *E. coli* MG1655 ancestor (Genbank accession number: U00096.3) and deleted five REPIN copies in a tandem cluster of six REPINs. From these numbers, we can estimate the magnitude of the amplification rate *λ* the same way Lee *et al.* have done for the substitution rate. To focus on the rate per REPIN we have to also divide by the REPIN population size of *E. coli* MG1655 (224) to obtain a maximum likelihood estimate of $\lambda =1/(6160\times 50\times 224)\approx 1.45\times 10^{-8}$. The 95% confidence interval of *λ* ranges from $9\times 10^{-10}$ to $6.38\times 10^{-8}$.

### REPIN transposition rate δ

Note, that strictly speaking, the REPINs we identify in *E. coli* and other enterobacterial strains are REP sequences. REPINs consist of two REP sequences in an inverted orientation. However, since REPINs in enterobacteria are asymmetric (*i.e.*, the 5′ REP sequence differs from the 3′ REP sequence by a single nucleotide deletion/insertion), it is difficult to identify and analyze the whole REPIN (Bertels *et al.* 2017b). However, despite focusing our analyses on REP sequences in enterobacteria, we speak of REPINs as these are the actual mobile elements. REP sequences, when encountered as singlets (which is relatively rare) are immobile remnants of REPINs (Bertels and Rainey 2011b).

We first identified the most common 21–25 bp long sequences in ten different Enterobacterial strains to determine approximate REPIN transposition rates. We identified the corresponding REPIN populations for each of these highly abundant sequences by recursively searching all sequences that differ in exactly one position from any already identified sequence in the genome [see Bertels *et al.* (2017b) for more details]. Using the mutation-selection (or Quasispecies) model, we inferred REPIN transposition rates as described in Bertels *et al.* (2017b). This model considers four mutation classes. The first mutation class only contains a single sequence, the master sequence. The second and third mutation classes contain all sequences that differ from the master sequence in exactly one and two positions, respectively. The last mutation class contains all sequences that differ in three or more positions to the master sequence. By assuming that the frequency distribution of the four mutation classes is in a steady-state, the REPIN transposition rate can be estimated for a constant mutation rate. Using this procedure, we obtained five transposition rates for the master sequence per bacterial strain, one for each sequence length. For each strain, we report the highest master sequence transposition rate. All estimated transposition rates are summarized in Table 1.

**Table 1:** Estimated transposition rates and REPIN population sizes

Strain	Seq.	Transp.	REPIN
	Length (bp)	Rate (δ)	Pop. size (*r*)
*Salmonella enterica* ATCC 9150	24	$5.2\times 10^{-9}$	98
*Citrobacter koseri* ATCC BAA-895	24	$3.8\times 10^{-9}$	323
*Enterobacteriaceae bacterium* FGI57	21	$9.3\times 10^{-9}$	150
*Klebsiella variicola* 342	23	$5.4\times 10^{-9}$	91
*Escherichia albertii* 07-3866	24	$7.6\times 10^{-9}$	226
*Escherichia coli* K-12 MG1655	23	$1.2\times 10^{-8}$	224
*Escherichia coli* B REL606	24	$9.7\times 10^{-9}$	220
*Escherichia coli* UMN026	21	$1.4\times 10^{-8}$	159
*Escherichia coli* UTI89	24	$7.7\times 10^{-9}$	137
*Escherichia coli* 536	24	$8.7\times 10^{-9}$	158

### Model

Our main objective is to explore the conditions that would allow REPINs to persist in their bacterial host genome for millions of years or billions of bacterial generations. We begin by describing the dynamics of the hosts—the bacteria. We assume that bacteria grow near exponentially when the population size is small, and growth saturates when the population size is close to carrying capacity (*i.e.*, logistic growth).

(1) $$\dot{B}=gB,$$

where *B* is defined as B=n/K, *K* is the population carrying capacity, and *n* is the number of bacteria in the population. *g* is defined as g=1−B.

We can define bacterial subpopulations depending on the number of REPINs *r* each bacterium carries. The relative abundance of bacteria carrying *r* REPINs with respect to *K* is denoted by $b_{r}=n_{r}/K$. The bacterial pool is the sum of all bacteria with different numbers of REPINs, $n=\sum_{r}n_{r}$. Hence *B* becomes

(2) $$B=\sum_{r}b_{r}.$$

**Figure 3:**
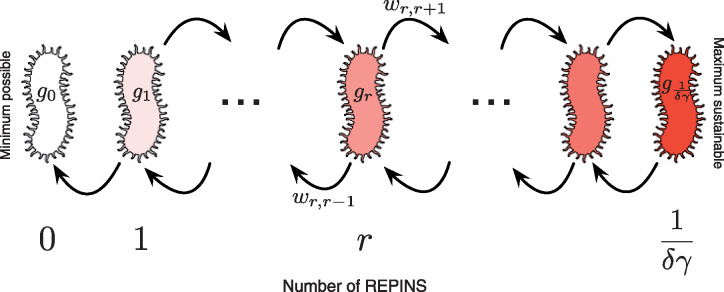
Modeling intragenomic sequence population in a bacterial population. Bacteria in the population only differ in the number of REPINs they contain. A bacterium with *r* REPINs gains a REPIN with rate $T_{r,r+1}$ and loses a REPIN with rate $T_{r,r-1}$. The gain and loss of REPINs depend on the parameter *λ* (random amplification and deletion of REPINs) and *δ* (REPIN transposition rate). The transposition rate *δ* also decreases the growth rate of each bacterium by rδγ, since with probability *γ* a bacterium will be killed after a transposition event. The minimum number of REPINs is zero, the upper REPIN population size limit for maintaining a viable bacterial population is given by r=1/(δγ).

The number of bacteria carrying *r* REPINs can change due to bacterial growth and the REPIN dynamics. For example, if a REPIN is deleted, the bacterium changes its state from *r* to *r—*1, which happens with rate $T_{r,r-1}$. Similarly, if a REPIN successfully duplicates then we see the transition from *r* to *r *+* *1, which happens at rate $T_{r,r+1}$. The REPIN dynamics are sketched in Figure 3. Altogether, the change in the relative bacterial abundance is captured by the following set of differential equations,

(3) $$\begin{array}{c} \dot{b_{r}}(t)=g_{r}b_{r}\\ +(T_{r-1,r}b_{r-1}+T_{r+1,r}b_{r+1})\\ -(T_{r,r-1}b_{r}+T_{r,r+1}b_{r}). \end{array}$$

Since having zero REPINs is a boundary condition, for *r *=* *0 we have $T_{r-1,r}=T_{r,r-1}=T_{r,r+1}=0$. The last equality also confirms that once the REPINs are lost, they cannot be regained.

We connect growth and transition rates in the above equation with our observation in the previous section. The RAYT transposase duplicates REPINs by copying them into another location of the genome (Bertels and Rainey 2011b). This transposition rate is denoted as *δ*. However, transposition comes at a cost. Once a REPIN is copied into a gene, then the gene will be destroyed. If the gene is essential for bacterial survival, then the bacterium that carries the REPIN population, including the transposed REPIN, will die. We denote *γ* as the fatality probability that a bacterium dies due to a REPIN transposition. Hence bacterial growth rate, *g_r_*, can be written as

(4) $$g_{r}=1-B-r\delta \gamma .$$

Our observation of REPINs in bacterial genomes suggests that besides the RAYT transposase activity, REPINs may be able to reproduce locally. Local amplification and deletion of REPINs are probably mediated by the host replication machinery and not by the RAYT transposase (Bertels and Rainey 2011a,b). This mode of amplification and deletion is captured by including a birth rate *λ* and an equal death rate *λ* giving the transition rates,

(5) $$\begin{array}{c} T_{r,r+1}=r[\lambda +\delta (1-\gamma )],\\ T_{r,r-1}=r\lambda . \end{array}$$

## Results

### Simple replicating intragenomic sequence populations cannot persist in bacterial genomes

**Figure 4:**
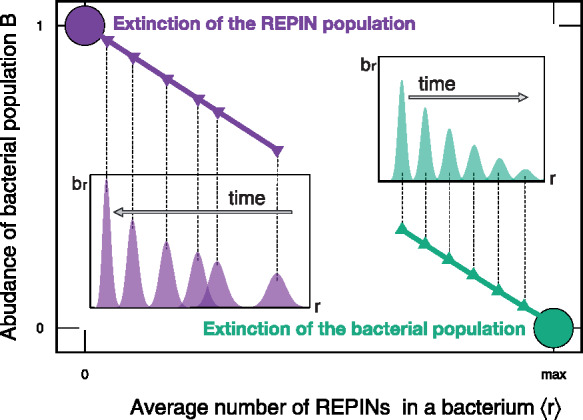
The dynamics of the bacterial pool and the average REPIN numbers found per bacterial genome under the base model. The *y*-axis shows the relative bacterial abundances $b_{r}/B$. The cartoon demonstrates the two possible stable equilibria of the bacterial population governed by Equation (3) with Equations (4) and (5). Different initial conditions lead to two different outcomes indicated by the purple and green circles. When the initial condition is close to the zero-REPIN state, the bacterial population follows the thick purple line leading to the extinction of the REPIN population. When starting with a large initial REPIN population size with a small fatality probability *γ*, the REPIN population size will increase across the entire bacterial population. Consequently, the bacterial population size will decrease and eventually go extinct (green line). Each point marked with an arrow shows the distribution of the bacterial population *b_r_* at that time point.

Our model describes a bacterial population in which each bacterium carries a certain number of REPINs *r*. REPINs can transpose to a different position in the genome through duplication. Every REPIN transposition can harm the bacterium. There is a chance *γ* that a REPIN transposition leads to the bacterial host’s death. This model will lead to two different outcomes depending on the parameter values and initial conditions. Either the REPIN population will go extinct in the bacterial population ($b_{0}=1$, purple distribution in Figure 4) or the REPIN population will grow uncontrolled and eventually drive the bacterial population to extinction (*B *=* *0, green distributions in Figure 4).

For a fatality probability greater than 0 (γ>0) any transposition event can lead to the death of the bacterial host, and thus the fittest subpopulation is the population without REPINs. Bacteria devoid of REPINs have the highest growth rate. They cannot acquire REPINs in the absence of *hgt*. Hence, as soon as a fraction of bacteria loses all REPINs, REPINs will go extinct in the bacterial population. REPIN extinction usually occurs when a population starts with small REPIN numbers or a large fatality probability (*γ*). When *γ* is large, bacteria are more likely to die after a transposition event than to successfully increase the REPIN number (purple distribution in Figure 4).

Alternatively, the accumulation of REPINs can lead to the extinction of the bacterial population. The bacterial population will go extinct when large REPIN numbers accumulate, for example, when the fatality probability (*γ*) is low (the bacterium is unlikely to die after a transposition event). In this case, an increasing number of REPINs will lead to a decreasing number of bacteria. Thus eventually the entire population becomes extinct (green distribution Figure 4).

We analytically prove that these two trivial scenarios are the only possible, stable solutions of our model (see Appendix B for detailed calculations), showing that our model agrees with existing literature. Hence, our basic model does not explain what we observe in nature: an intragenomic sequence population that persists for millions of years.

### Horizontal gene transfer within a bacterial population cannot explain REPIN persistence

*Hgt* has been shown to be essential to explain the persistence of selfish genetic elements (Doolittle and Sapienza 1980; Sawyer and Hartl 1986; Bichsel *et al.* 2010). Although for REPINs there is no evidence of significant *hgt*, at least on the species level (Bertels *et al.* 2017a), *hgt* within populations may be able to explain the persistence of REPINs as shown for a specific model and a very specific parameter set in ISs (Bichsel *et al.* 2013).

To understand how exactly *hgt* affects the evolutionary dynamics of REPIN populations, we implemented *hgt* as a simple mixing process to mimic the process of gene conversion (Vos 2009). Currently, we believe that replicative *hgt* is unlikely to occur for REPINs, since they are nonautonomous elements and cannot simply copy themselves on a plasmid and then from that plasmid back into a new host unless the RAYT gene is copied at the same time. Furthermore, RAYT genes have not been observed on plasmids compared to IS elements, and do not copy themselves (Bertels *et al.* 2017a).

The *hgt* rate *h* determines the frequency at which REPINs are transferred from one bacterium to another.

(6) $$\begin{array}{c} T_{r,r+1}=r[\lambda +\delta (1-\gamma )]+\frac{h}{B}\sum_{r}rb_{r},\\ T_{r,r-1}=r(\lambda +h). \end{array}$$

This mixing process makes the complete loss of REPINs (*b*_0_) reversible, allowing bacteria without REPINs to gain a REPIN from the rest of the population.

However, even though *hgt* provides a way to escape the zero-REPIN state, *hgt* by itself does not lead to a sustainable REPIN population. The number of REPINs in the population will still either decrease until all bacteria lose all REPINs or increase until the bacterial population is extinct.

Whether the REPIN population or the bacterial population goes extinct is mainly determined by the fatality probability *γ* for high *hgt* rates (Appendix C). For γ<0.5 REPIN population size increases to infinity because REPINs successfully duplicate most of the time (eukaryotic regime in Figure 1). In contrast, REPINs go extinct for γ>0.5 due to a twofold effect: (1) REPIN populations grow more slowly because most transposition events are unsuccessful and (2) carrying REPINs is more costly because transposition events often kill the bacterial host (Prokaryotic regime in Figure 1). Hence, as established previously with similar models, *hgt* alone cannot stabilize a REPIN population in bacterial genomes.

### Beneficial effects can lead to stable REPIN population sizes

To explain the persistence of REPINs in the genome, we propose a mutualistic relationship between REPINs and their host. In a simple model, each REPIN contributes a constant benefit *α* to the host. The total fitness benefit will then be *αr*. Besides being unrealistic (adding too much of anything will eventually be detrimental), such a benefit function does not lead to a stable REPIN population. If *α* is smaller than the transposition rate *δ*, then the possible steady states do not change; either REPINs get purged from the genome, or the whole bacterial population goes extinct together with the REPINs. If *α* is larger than *δ*, then REPIN population size will grow to infinity and so will the bacterial population size, which is not a plausible scenario.

Ergo the fitness benefit function needs to be more complex to describe a realistic biological scenario. An additional parameter, *w* modifies the beneficial effect each additional REPIN provides to the host. The following functional form changes the benefit provided by each additional REPIN, where *w* is the base of the change: (Dawes *et al.* 1986; Hauert *et al.* 2006; Gokhale and Hauert 2016),

(7) $$\begin{array}{c} \Gamma (r)=\alpha +\alpha w+\alpha w^{2}+\alpha w^{3}+\cdots +\alpha w^{r-1}\\ =\alpha \frac{\left(1-w^{r}\right)}{1-w}. \end{array}$$

**Figure 5:**
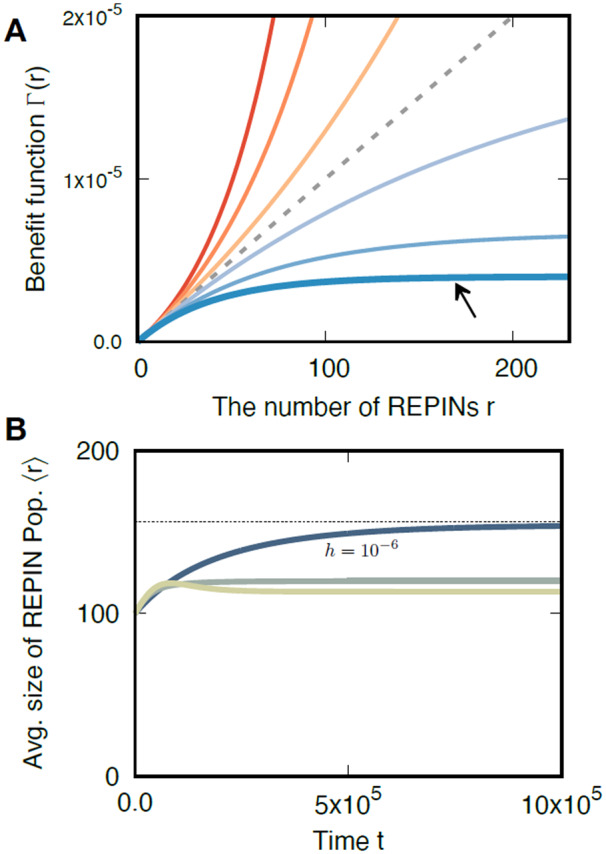
Benefit functions and dynamics of average REPIN numbers 〈r〉 for various *hgt* rates. (A) Benefit function with synergy (*w *>* *1) and discounting (*w *<* *1) effects. Total benefit Γ(r) increases with the number of REPINs *r*. With *w *=* *1 the benefit a REPIN provides is constant (gray dashed line). For *w *>* *1, REPIN benefits are synergistic, *i.e.*, each additional REPIN provides a greater benefit than the previously added REPIN. For *w *<* *1, REPIN benefits are discounting, *i.e.*, each additional REPIN provides a smaller benefit than the previously added REPIN. The black arrow points at the benefit function, which is used in (B). (B) Changes of average REPIN population sizes 〈r〉 over time for different *hgt* rates (*h*). The black dotted line is the expected REPIN population size (〈r〉) at the steady-state for high *hgt* rates. Lower *hgt* rates lead to smaller average REPIN population sizes. We used the following model parameters $\gamma =0.55,\;\delta =\lambda =10^{-8},\;\alpha =5\times 10^{-8}$, and *w *=* *0.975.

The benefit function Γ(r) captures the total benefit of *r* REPIN sequences (Figure 5A). For *w *=* *1 each REPIN provides a constant benefit *α* (discussed above). With *w *<* *1, each additional REPIN provides a smaller benefit, saturating the total benefit. Similarly, with *w *>* *1, each additional REPIN provides a larger benefit, exponentially increasing the total benefit. The beneficial effect of REPINs is reflected in the bacterial growth rate, $g_{r}=1-B-r\delta \gamma +\Gamma (r)$.

Decreasing benefits (*w *<* *1) allow a stable REPIN population to persist in the bacterial genome (Figure 5B). For high *hgt* rates, we can analytically determine the size of the REPIN population in steady-state. To obtain a stable REPIN population, the fatality rate needs to be high (γ>0.5) and the benefit strength *α* needs to be higher than δ(2γ−1) (Appendix D for the detailed calculation). For these conditions, we can calculate the average number of REPINs in a bacterial genome:

(8) $$\left\langle r\right\rangle =\frac{1}{1-w}\ln\left(\frac{\alpha }{\delta (2\gamma -1)}\right).$$

A careful analysis of the model parameters shows that few parameter combinations yield a REPIN population of biologically relevant size. The REPIN population size is determined by three free parameters (*α*, *γ* and *w*). We set the parameter range for *α* to $10^{-7}-10^{-9}$, close to the transposition rate *δ*, also determining the fitness cost of each REPIN. The other two parameters are bounded by the model itself: *γ* can range from 0.5<γ<1 and *w* can range from 0<w<1.

**Figure 6:**
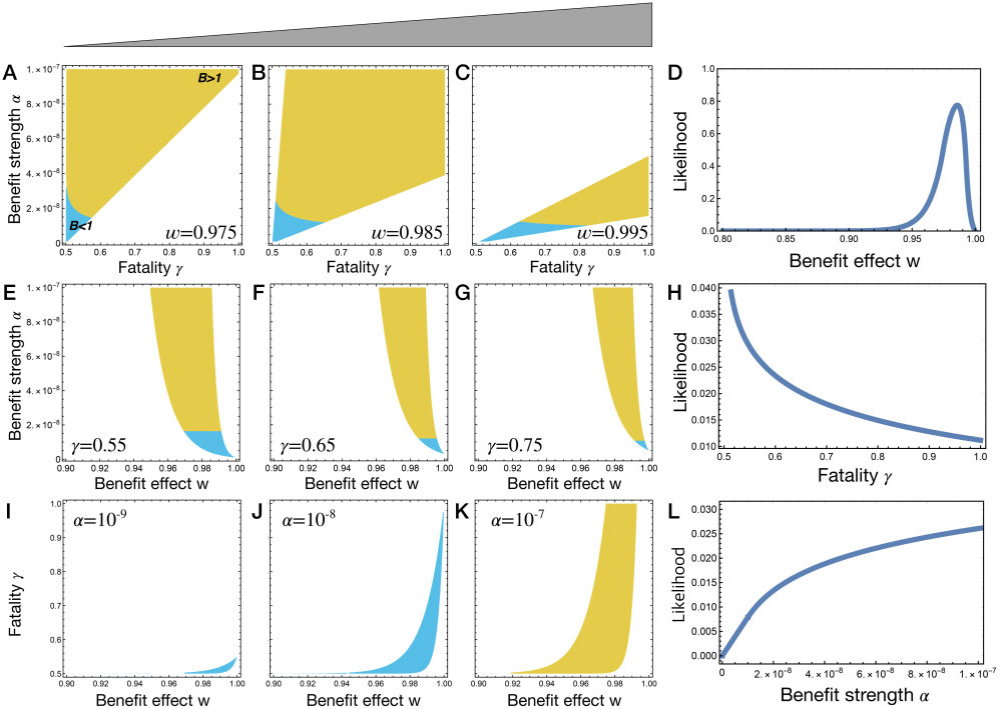
Observable parameter range and likelihood. Three model parameters, benefit effect *w*, fatality probability *γ*, and benefit strength *α*, determine the average REPIN population size 〈r〉. Only certain parameter combinations result in biologically relevant REPIN population sizes (*i.e.*, between 91 and 323 REPINs, Table 1). To visualize this observable parameter range, we fixed one parameter while the other two parameters varied. In the colored area, REPIN population sizes are between 91 and 323. The carrying capacity *K* is measured in the absence of REPINs. Hence, the bacterial population size increases with REPINs in yellow-colored areas, while bacterial population size becomes smaller in blue-colored areas. Each row is associated with one parameter. For example, in the first row, for three fixed benefit effects *w*, we determine the observable parameter range (A–C). The size of the observable parameter range is plotted in (D), which corresponds to the likelihood that a *w*-value is part of a parameter combination that leads to a stable REPIN population of observable size. The second and third rows show the same plots for the fatality probability *γ* and benefit strength *α*, respectively. Note that for all parameter ranges (0<w<1, 0.5<γ<1, and $10^{-9}\leq \alpha \leq 10^{-7}$) the proportion of parameter combinations that result in stable REPIN populations of observable size is only about 2%.

Each parameter combination yields an average REPIN population size in the bacterial population. Yet, the biologically relevant REPIN population sizes should be between 91 and 323 REPINs (Table 1). To assess, which parameter combinations lead to biologically relevant REPIN population sizes one of the three free parameters was fixed. The other two parameters were varied across the entire range (Figure 6).

Without a detailed analysis of our model, the biologically relevant range of the discounting effect *w* (how strongly the benefit of each REPIN decreases with increasing REPIN number) is hard to predict. However, our model suggests that the effect needs to be in the range of 0.95 and 0.99 (Figure 6D). Otherwise, the other parameter values have to become unrealistic to yield suitable average REPIN population sizes. Intuitively, this means that the host’s benefit decreases by 1–5% with each REPIN added to the genome. Furthermore, for large discounting effects, relevant REPIN population sizes are only observed for small fatality probabilities (Figure 6A). On the contrary, small discounting effects require a small benefit strength to lead to relevant REPIN population sizes (Figure 6C).

In our model, it is impossible to maintain a stable REPIN population if *γ* is below 0.5 because this regime would lead to an ever-increasing sequence population. Hence, at least 50% of the bacterial genome needs to be critical for long-term survival to maintain REPIN populations. A fatality probability of close to 0.5 also yields the most parameter combinations to maintain a stable REPIN population (Figure 6H).

Finally, high-benefit strength is most likely to yield a stable REPIN population (Figure 6, I–K). Whereas low benefit strength $\left(10^{-9}\right)$ is only possible when the discounting effect is close to 1 and the fatality probability is close to 0.5 (Figure 6I) and always leads to bacterial populations that are less fit than a population without REPINs.

## Discussion

In prokaryotic genomes, TEs get continuously purged from the genome due to a combination of low *hgt* (and recombination rates) and the high cost of transposition. As a result, ISs are usually present in only a fraction of strains within a species (Touchon and Rocha 2007). Nonautonomous REPINs are different. If present in a species, then most strains of that species will contain a significant number of REPINs. To maintain a large number of TEs inside a genome, where transposition costs are high and *hgt* is low, the continuous extinction process has to be halted.

Here, we propose a model that endows each REPIN with a fitness benefit to the host bacterium. The benefit function prevents the REPIN population from going extinct and allows them to be maintained as a stable population inside the bacterial genome. The benefit, however, follows a particular functional form. If each REPIN provides a constant beneficial effect to the bacterium, REPIN populations are still not stably maintained. If the benefit is lower than the cost of carrying a REPIN, then the aforementioned scenarios apply, otherwise the REPIN and bacterial population size increase indefinitely. Only discounting benefits (*i.e.*, the benefit each REPIN provides decreases with increasing REPIN population size) can lead to stable REPIN population sizes.

In eukaryotes, sequence populations have been discovered and modeled since in the 1980s (Hickey 1982; Charlesworth and Charlesworth 1983; Charlesworth and Langley 1989). The models suggest that instead of preventing TEs from going extinct, TEs have to be prevented from indefinitely accumulating in the genome in eukaryotes. Accumulation can be stopped when the cost of carrying TEs increases synergistically or the transposition rate is regulated. Interestingly, a synergistic increase of fitness costs, in eukaryotes, is a symmetric solution to discounting fitness benefits in prokaryotes (Figure 1). The reason for this symmetry probably lies in the cost of transposition. In prokaryotes, transposition is very costly (γ>0.5), and hence extinction needs to be prevented by supplying a benefit, whereas in eukaryotes the low cost (γ<0.5) of transposition leads to increasing TE population sizes that have to be countered by a synergistically increasing fitness cost, eventually pushing the fatality rate *γ* in our model past 0.5. In both cases, the TEs modify host fitness to form stable sequence population sizes.

Our results also show that only a small subset of discounting fitness functions allow REPINs to persist in bacteria. The range is particularly small for the discounting effect *w*. Only if the benefit each additional REPIN provides decreases by about 1% to 5%, are there many parameter combinations that lead to a REPIN population of biologically relevant size (*i.e.*, between 91 and 323 REPINs). The surprisingly narrow range of the discounting effect will allow us to test our model in the future. In a laboratory experiment, one could, for example, delete all REPINs in a single bacterial strain (*e.g.*, with CRISPR technology) and then add REPINs one at a time (or vice versa). We would expect the average additional benefit for each REPIN added to decrease by about 1–5%.

The fitness advantage of bacteria carrying a single REPIN over bacteria carrying no REPINs should be on average in the range of the benefit strength *α*. The benefit strength *α* is expected to be low per individual REPIN $\left(10^{-9}<\alpha <10^{-7}\right)$. Low benefit strength is a consequence of low levels of harm done by REPIN transposition due to low-transposition rates $\left(\;10^{-8}\right)$. Interestingly, even when the benefit provided by each REPIN is less than the harm done (α<δ) it is possible to maintain stable REPIN populations at least in the presence of *hgt*. It is unclear whether these results still hold in the absence of *hgt*, which might be more biologically relevant. Our simulations suggest that in almost all cases where bacterial populations survive with low benefits in the presence of *hgt*, the populations would not survive in the absence of *hgt* (Appendix D).

Currently, there is little evidence to what benefit REPINs (in conjunction with RAYTs) could provide to the host bacterium. We have previously speculated that RAYTs and REPINs could be part of a promoter (REPIN) and transcription factor system (RAYT) (Bertels and Rainey 2011a). This speculation was based on the fact that REP sequences (repetitive components of REPINs) have been shown to affect gene expression of neighboring genes by terminating transcription or affecting mRNA half-life (Merino *et al.* 1987; Espéli *et al.* 2001; Liang *et al.* 2015). In turn, the RAYT protein could modify this effect by binding to the REPIN and excising it from the mRNA. Alternatively, RAYT and REPINs could affect gene expression by altering folding of the DNA; another function REP sequences have been implicated in Yang and Ames (1988) and Qian *et al.* (2015).

For ISs, it has been argued that they can increase their persistence time because they occasionally cause beneficial mutations in the host (Schneider *et al.* 2000; Startek *et al.* 2013). It is unlikely that the same argument can be made for REPINs, for the following reasons. First, REPINs are maintained for millions of years as a stable sequence population. If it were possible to explain their persistence through occasional beneficial mutations, then we would also expect IS elements to persist as populations, which they do not. Second, one of the reasons IS elements cannot persist over long periods inside bacterial genomes, is that the mutator phenotype they can cause is extremely costly (every additional insertion increases the transposition rate and hence also the mutation rate), unable to compete with mutator phenotypes generated through mutations in *mut* genes (Wielgoss *et al.* 2013; Consuegra *et al.* 2021). Third, to significantly contribute to the host bacterium’s mutation rate, REPIN transposition rates would have to be 1000 times higher than measured in *E. coli* and other species (Bertels *et al.* 2017b).

Another appealing aspect of our study is the result concerning the fatality probability *γ*. The fatality probability describes what proportion of REPIN transposition events leads to the death of a bacterium. Our model suggests that *γ* has to be larger than 0.5 to yield a stable REPIN population. This result was somewhat surprising to us and initially did not seem to be compatible with the biological reality since studies have shown that only about 10% of all genes in the genome are essential (Baba *et al.* 2006; Freed *et al.* 2016). For fatality probabilities of less than 0.5 it is impossible to maintain sequence populations in bacterial genomes under our model.

One could also argue that *γ* describes the proportion of essential genes in at least one of the bacterium’s natural environments. That the set of essential genes in one environment differs from essential genes in a different environment has been shown in *E. coli* (Nichols *et al.* 2011). Hence, if *E. coli* regularly encounters a large range of different environments; the proportion of genes that contribute to fitness that is too high to result in their loss from the genome might exceed 50%.

Another indicator of the importance of genes for long term survival is the size of the core genome. In *E. coli* about 46% of the genes in the genome are found in all strains (Touchon *et al.* 2009). Suppose we now add essential noncoding regions such as rRNa, tRNA, and essential regulatory regions. In that case, it is likely that indeed more than 50% of the genome is essential for long-term survival of the strain. What “long term” means depends, of course, on how we define a species. The most common ancestor of all *E. coli* strains is predicted to have lived about 15 million years ago (Ochman *et al.* 1999; Bertels *et al.* 2017b). Hence, about 50% of the genes were necessary for all *E. coli* strains to survive for the last 15 million years. For a more specialized subset of strains within the *E. coli* species a much larger proportion of genes is expected to be shared and important for survival. Hence, it seems plausible that a large proportion of the bacterial genome is required for long-term survival as predicted by our model. If this is not the case, then the number of repetitive sequences should increase over time, similar to what can be observed in birds and mammals, where transposon replication is only counteracted by infrequent loss events of large parts of the genome (Kapusta *et al.* 2017).

Our results in Figure 6 only hold if the *hgt* rate is much higher than the transposition rate *δ*. Active *hgt* mediated by the RAYT transposase is very unlikely to occur in nature (Bertels *et al.* 2017a). Although REPINs and RAYTs may be passively transferred to other genomes through homologous recombination (Guttman and Dykhuizen 1994), the resulting REPIN transfer rate is probably low. Hence, the results in Figure 6 might not be directly applicable to REPIN populations. Nevertheless, simulations for low-*hgt* rates show that REPIN populations can persist without *hgt*, given that the REPIN population is beneficial for the host (Appendix D).

In the absence of *hgt* antagonistic coevolution as observed for other mobile genetic elements is nigh impossible. A predominantly vertical mechanism of inheritance ties the evolutionary fate of REPINs almost entirely to the host’s fate. The only way to ensure REPIN survival is to ensure the survival of the host. REPIN populations that are not providing enough of a benefit will be purged. Hence, coevolution between REPINs and the bacterial host is unlikely to be antagonistic compared to other mobile genetic elements.

One of the main issues we have not addressed in our current study is the RAYT transposase evolution. If we assume that RAYTs can be lost and gained from the genome leading to a REPIN transposition rate *δ* of 0, then our model’s long-term dynamics could change. Extending our current model with the possibility of RAYT evolution requires at least one more parameter to describe RAYT gain and loss rates. In Appendix E, we present an elementary analysis of such a model. We assume that the number of RAYTs linearly increases the transposition rate *δ* but does not affect the benefit accrued by the REPIN (an assumption ripe for experimental testing). A numerical simulation of the extended model shows that REPINs are maintained at a stable equilibrium, which slightly varies between bacteria containing a RAYT gene(s) and bacteria that do not contain a RAYT gene. We plan to extend this model in the future to set RAYT gain and loss rates to correspond to observed data. Ideally, such a model may accurately predict a set of parameters that could, for example, explain the *E. coli* data presented in Figure 2.

Currently, all our analyses are deterministic. Although these models do not currently allow us to measure the long term stability of the system, we are confident that at least for REPIN populations that are larger than 100 individuals, populations should be stable for long periods [as investigated in a previous study (Bertels *et al.* 2017b)]. Hence, the conditions explored here could explain the presence and maintenance of sequence populations in bacterial genomes.

In conclusion, our analyses show that discounting beneficial effects can explain the presence of stable REPIN populations in bacterial genomes. The small parameter range of our benefit function provides a plethora of testable hypotheses on the evolution of intragenomic sequence populations in bacterial genomes.

## Data Availability

All codes with code README file for simulations are available on GitHub. Data for Figure 1 are in the same repository in a subfolder REPINSDataFig.

## Figures and Tables

**Figure C1:**
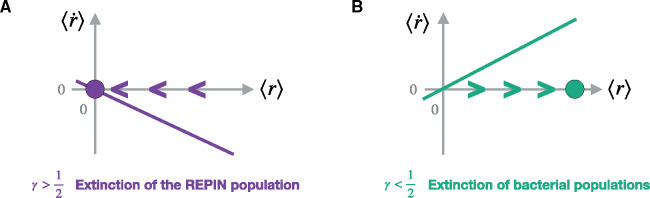
Phase portrait with high *hgt* rates. (A) For γ>0.5 the number of REPINs decreases to zero. (B) For γ<0.5 the number of REPINs increases without any bound, decreasing the bacterial pool *B*. Thus, the whole bacterial population as well as REPINs go extinct.

**Figure D1:**
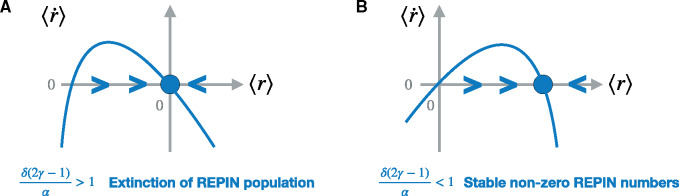
Phase portrait with high *hgt* rates and discounting beneficial effects for γ>0.5. For γ>0.5, duplication decreases REPIN numbers. Hence, we need a high benefit to overcome this driving force. (A) For δ(2γ−1)>α the number of REPINs decreases to zero. (B) For δ(2γ−1)<α a stable REPIN population can stably exist in the bacterial genome. This is even possible when the REPIN population decreases bacterial fitness.

**Figure D2:**
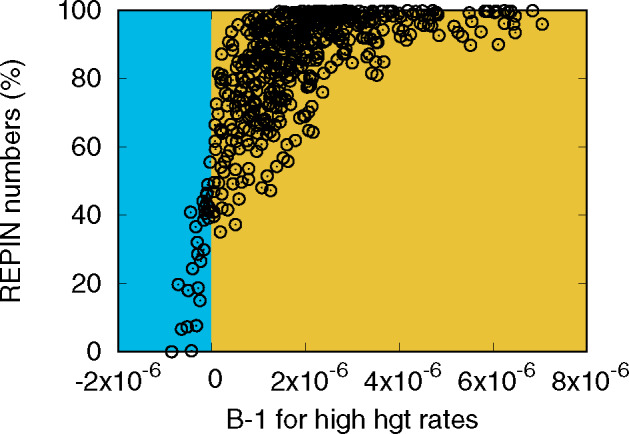
We randomly draw 500 parameter sets, which yield biologically observable REPIN numbers (91≤〈r〉≤323). Then, we numerically obtain REPIN numbers for *h *=* *0. *Y*-axis shows the ratio between REPIN population sizes obtained without *hgt* and population sizes with high *hgt*. Hence, 100% indicates that the REPIN population size without *hgt* is the same as the one calculated for high *hgt*, meaning the expression in Equation (28) is a good estimation. The extinction of REPINs is shown as 0%. When carrying a REPIN population is beneficial for the bacterium, the results calculated for high *hgt* rates are similar to numbers obtained without *hgt*. Especially when *B *<* *1, REPIN population sizes are significantly lower in the absence of *hgt*.

**Figure D3:**
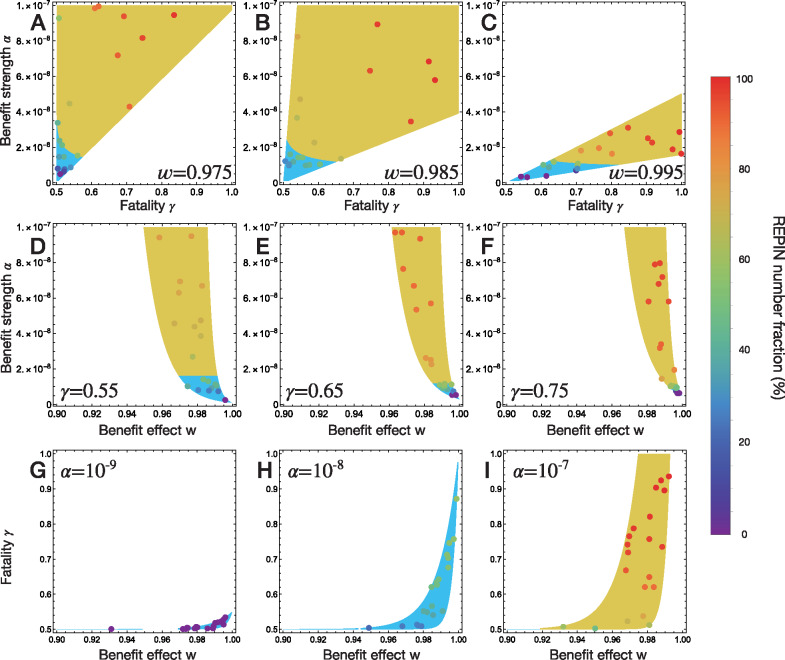
To clearly show that REPIN numbers abruptly drop when *B *<* *1, we randomly sampled 20 points in each panel in Figure 4 of the main text. If there are both regimes *B *>* *1 and *B *<* *1 in a single panel, we sampled 10 parameter sets for each regime. Otherwise, we sample all 20 points in one regime. We again use the same definition of REPIN number fraction in Figure 9 to show how much REPIN numbers can be achieved compared to expected values for high *hgt* rates at given parameter sets. Points in each panel show sampled parameter set and color indicate REPIN number fraction. As we can see, when *B *<* *1 (blue colored region) is expected for high *hgt* rates, the REPIN number fractions are low. On the contrary, for *B *>* *1 (yellow-colored region), REPIN number fractions can reach 100%.

**Figure E1:**
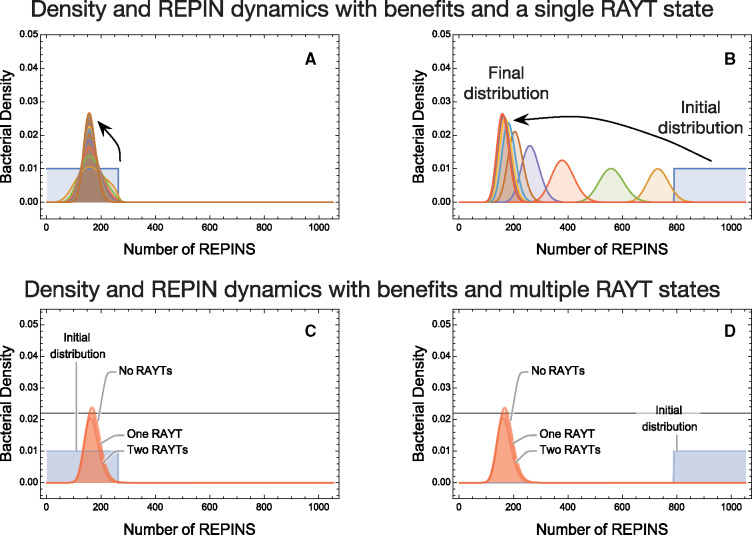
The extended model described by Equation (35) is implemented. We set *l *=* *1 for all bacteria as an initial condition. For *ϵ*  =  0 the model reproduce the original model, (A and B). With benefit effect, $\alpha =10^{-2}$ the nonzero REPINs can sustain with different initial REPIN distributions. For ϵ=0.2 value, nonzero REPINs still remain, (C and D). Three distributions in (C and D) indicate the REPIN distribution at a given RAYT numbers *l*. The other parameter values are γ=0.95 and the synergy/discounting value *w *=* *0.985.

## Appendices

### A Dynamics of the average number of REPINs

Here, we calculate the dynamics of the average number of REPINs, $\dot{\langle r\rangle }$,

(A1) $$\begin{array}{c} \dot{\langle r\rangle }=\sum_{r=0}^{\infty }r\dot{\left(\frac{b_{r}}{B}\right)}\\ =-\delta \gamma \langle r^{2}\rangle +\delta \gamma {\langle r\rangle }^{2}\\ +\sum_{r=1}^{\infty }[\lambda +\delta (1-\gamma )][r(r-1)b_{r-1}-r^{2}b_{r}]/B+\sum_{r=1}^{\infty }\lambda [r(r+1)b_{r+1}-r^{2}b_{r}]/B. \end{array}$$

For the first summation, we obtain

(A2) $$\begin{array}{c} \sum_{r=1}^{\infty }[\lambda +\delta (1-\gamma )][r(r-1)b_{r-1}-r^{2}b_{r}]/B=[\lambda +\delta (1-\gamma )]\sum_{r=0}^{\infty }[(r+1)rb_{r}-r^{2}b_{r}]/B\\ =[\lambda +\delta (1-\gamma )]\langle r\rangle , \end{array}$$

and for the second summation,

(A3) $$\begin{array}{c} \sum_{r=1}^{\infty }\lambda [r(r+1)b_{r+1}-r^{2}b_{r}]/B=\lambda \sum_{r=0}^{\infty }[(r-1)rb_{r}-r^{2}b_{r}]/B\\ =-\lambda \langle r\rangle . \end{array}$$

Altogether, we obtain the expression

(A4) $$\dot{\langle r\rangle }=\delta [\gamma \left({\langle r\rangle }^{2}-\langle r^{2}\rangle \right)+(1-\gamma )\langle r\rangle ].$$

Duplication can decrease the number of REPINs by killing the host bacterium. On the other hand, successful duplication leads to an increase of REPINs. The REPIN number can increase even when this leads to lower host fitness. Precisely these two decreasing and increasing forces of REPIN numbers are reflected in the first and second terms in Equation (A4), respectively.

### B Trivial solutions: extinction of REPINs or extinction of both REPINs and bacteria

In this section, we will show that only trivial solutions are achieved without *hgt* and beneficial effects. For convenience, we convert the relative abundances *b_r_* into fractions $f_{r}=b_{r}/B$. Then, the main equations become,

(B1) $$\begin{array}{c} {\dot{f}}_{r}(t)=\delta \gamma \left(\langle r\rangle -r\right)f_{r}+[\lambda +\delta (1-\gamma )][(r-1)f_{r-1}-rf_{r}]+\lambda [(r+1)f_{r+1}-rf_{r}],\\ \dot{B}=B\left(1-B-\delta \gamma \langle r\rangle \right), \end{array}$$

with *f_r_* = 0 for *r *<* *0. From the above equation, we can obtain *f*_1_ in steady-state as $f_{1}=-\frac{\delta \gamma \langle r\rangle }{\lambda }f_{0}$. The result indicates that *f*_1_ must be zero since negative values are forbidden for *f_r_*. Hence, either 〈r〉=0 or $f_{0}=0$ must be satisfied. Then the possible solutions are the extinction of REPINs ($f_{0}=1$ and *f_r_* = 0 for *r *>* *0) or the extinction of both REPINs and bacteria (*b_r_* = 0 for all *r*).

Birth and death resulting from *λ* make the population diffuse in state space. If all bacteria reach the 1/(δγ)-REPIN state before any bacterium enters the zero-REPIN state, the bacterial population dies. Otherwise, REPINs will go extinct in the bacterial population, and only zero-REPIN bacteria remain. The above analysis holds at any *λ* values.

### C Equations of motion with *hgt*

*Hgt* can also make a bacterium loss or gain a REPIN. We assume that *hgt* happens within the population. In this case, the role of *hgt* is mixing REPINs between bacteria. No external source of REPIN is assumed to exist. With the *hgt* rate *h*, a bacterium loses REPINs proportionally to how many REPINs it contains. The insertion of REPINs can occur in any state independent of REPIN numbers. Hence, transition rates with *hgt* are given by

(C1) $$\begin{array}{c} T_{r,r+1}=r[\lambda +\delta (1-\gamma )]+\frac{h}{B}\sum_{r}rb_{r},\\ T_{r,r-1}=r(\lambda +h). \end{array}$$

Accordingly, we obtain

(C2) $$\begin{array}{c} {\dot{b}}_{r}(t)=g_{r}b_{r}\\ +[\lambda +\delta (1-\gamma )][(r-1)b_{r-1}-rb_{r}]\\ +(\lambda +h)[(r+1)b_{r+1}-rb_{r}]\\ +h\langle r\rangle (b_{r-1}-b_{r}). \end{array}$$

For frequencies *f_r_*, the equations become

(C3) $$\begin{array}{c} {\dot{f}}_{r}(t)=-\delta \gamma \left(r-\langle r\rangle \right)f_{r}\\ +[\lambda +\delta (1-\gamma )][(r-1)f_{r-1}-rf_{r}]\\ +(\lambda +h)[(r+1)f_{r+1}-rf_{r}]\\ +h\langle r\rangle (f_{r-1}-f_{r}),\\ \dot{B}=B\left(1-B-\delta \gamma \langle r\rangle \right). \end{array}$$

In the previous section, we found that only two trivial solutions are possible in the steady-state without *hgt*. In this section, we examine possible other steady-state solutions with *hgt*. Because we cannot obtain a general solution for any *h* values, we focus on the extreme cases first and then analyze the general case.

#### Low *hgt* regime

By solving for ${\dot{f}}_{0}=0$ from Equation (C3), we can obtain

(C4) $$f_{1}=\frac{\langle r\rangle (h-\delta \gamma )}{\lambda +h}f_{0}.$$

Hence, *f*_1_ should be zero for h≤δγ and accordingly all *f_r_* for *r *>* *1 also become zero. It means that only trivial solutions are possible for h≤δγ. The results remain the same for all possible *λ* values.

#### High *hgt* regime

By solving ${\dot{f}}_{0}=0$ from Equation (C3) with assumption λ≪h and δ≪h, we can get

(C5) $$f_{1}\approx \langle r\rangle f_{0}.$$

In the same way, we can recursively get the solutions,

(C6) $$\begin{array}{c} f_{2}\approx \frac{\langle r\rangle }{2}f_{1},\\ f_{3}\approx \frac{\langle r\rangle }{3}f_{2},\\ \vdots \\ f_{r}\approx \frac{\langle r\rangle }{r}f_{r-1}=\frac{{\langle r\rangle }^{r}}{r!}f_{0}. \end{array}$$

Because there is a relation between *f_r_* and 〈r〉, $\langle r\rangle =\sum_{r}rf_{r}$, we can get $f_{0}=e^{-\langle r\rangle }$. Thus, the final solution of the stationary distribution is

(C7) $$f_{r}=\frac{{\langle r\rangle }^{r}}{r!}e^{-\langle r\rangle }.$$

The resulting distribution does not depend on *h* value itself once the rate *h* is high enough.

For high *hgt* rates, the distribution is accessible so that we can calculate $\langle r^{2}\rangle$. Plugging Equation (C7) into Equation (A4), we found the dynamics of the average REPIN numbers.

(C8) $$\dot{\langle r\rangle }=\delta (1-2\gamma )\langle r\rangle .$$

For high fatality, γ>0.5, the REPIN population dies out because (1) bacteria carrying more REPINs are more likely to die than bacteria carrying fewer REPINs and (2) REPINs proliferate more slowly since most duplication are not successful (Figure C1A). For low fatality, γ<0.5, duplications are less harmful, and hence lead to an increase in REPIN numbers (Figure C1B). Even though it leads to lower bacterial fitness and the eventual extinction. Only exactly at γ=0.5 does REPIN population size 〈r〉 stay constant. However, this scenario is not biologically relevant because any perturbation of *γ* will lead to a population collapse.

If λ≪h is not guaranteed, the distribution *f_r_* at the steady-state becomes

(C9) $$f_{r}=f_{r-1}\frac{h\langle r\rangle +(r-1)\lambda }{r(h+\lambda )}.$$

Assuming λ≫h, we can get $f_{r}=f_{r-1}(r-1)/r$, which leads to $f_{1}=0$. Thus we again obtain the trivial solutions. But we are not sure whether the extinction of REPINs happen first or not according to *γ*.

#### Intermediate *hgt* regime

For two extreme cases, h≤δγ and λ≪h, we found that *hgt* cannot support the persistence of REPINs in the bacterial population. For intermediate *hgt*, δγ<h≲λ, we cannot apply the analytic approach, and thus we numerically investigate the possible solutions in this regime. As shown above, the solutions of *f_r_* at the steady-state can be recursively calculated from *f*_0_. For example, by solving ${\dot{f}}_{0}=0$, we can express *f*_1_ in terms of *f*_0_ and 〈r〉. In the same way, by solving ${\dot{f}}_{1}=0$, now we can express *f*_2_ in terms of *f*_1_, *f*_0_, and 〈r〉. Since *f*_1_ can be expressed by *f*_0_ and 〈r〉, *f*_2_ can be expressed by *f*_0_ and 〈r〉. In the same way, all *f_r_* can be expressed in terms of *f*_0_ and 〈r〉. Here, we numerically search for a possible set of *f*_0_ and 〈r〉 with two constraints: (1) $\langle r\rangle =\sum_{r=0}^{l}rf_{r}$ and (2) $\sum_{r=0}^{l}f_{r}=1$. Parameter sets γ∈{0.1, 0.3, 0.5, 0.7, 0.9} and $h\in \{5\times 10^{-9},10^{-8},5\times 10^{-8},10^{-7}\}$ with $\delta =\lambda =10^{-8}$ and the maximum REPIN population size *l *=* *50 are investigated. For all sets, only trivial solutions can be achieved, implying that *hgt* does not allow REPINs to persist.

### D Equations of motion with mutualism

Now we explore the regime where REPINs can have a positive effect on bacterial growth,

(D1) $$g_{r}=1-B-r\delta \gamma +\Gamma (r),$$

where the benefit function is

(D2) $$\Gamma (r)=\alpha \frac{\left(1-w^{r}\right)}{1-w}.$$

Then, the equations of motion become

(D3) $$\begin{array}{c} \dot{\langle r\rangle }=\langle r\Gamma \rangle -\langle r\rangle \langle \Gamma \rangle +\delta [\gamma \left({\langle r\rangle }^{2}-\langle r^{2}\rangle \right)+(1-\gamma )\langle r\rangle ]\\ \dot{B}=B\left[1-B-\delta \gamma \langle r\rangle +\langle \Gamma \rangle \right]. \end{array}$$

To understand the above equation, we should know the second moment of *r*, $\langle r^{2}\rangle$. Here, we use the backward Euler method to solve Equation (C2) with the growth rate denoted as Equation (D1). Again we will obtain the analytic results for the high *hgt* regime first.

#### High *hgt* regime

For the high *hgt* regime, α≪h, mixing of REPINs between bacteria happens fast enough, and thus the distribution *f_r_* becomes smooth with a single peak around the average REPIN numbers 〈r〉 showing the Poisson distribution again,

(D4) $$f_{r}=\frac{{\langle r\rangle }^{r}}{r!}e^{-\langle r\rangle }.$$

Now we can calculate $\langle r^{2}\rangle$ giving the solvable dynamics of 〈r〉,

(D5) $$\dot{\langle r\rangle }=\left[\alpha e^{-\langle r\rangle (1-w)}+\delta (1-2\gamma )\right]\langle r\rangle .$$

Solving the equation for equilibrium, we can obtain the average REPIN population size 〈r〉 at the steady-state,

(D6) $$r^{*}=\frac{1}{1-w}\ln\left(\frac{\alpha }{\delta (2\gamma -1)}\right).$$

In parallel, *B* at steady-state is

(D7) $$B=1+\frac{\alpha +\delta -2\gamma \delta -\gamma \delta \ln\left(\frac{\alpha }{\delta (2\gamma -1)}\right)}{1-w}.$$

Note that this solution is valid only for *w *<* *1 (discounting effect) and γ>0.5. From this estimation, we can find the possible parameter ranges of *α*, *γ*, and *w* to observe REPIN population sizes found in nature.

#### Stability analysis

Even if there is a fixed point for nonzero REPIN numbers, it could be unstable. In this case, a stable REPIN population cannot be maintained. Now, we will check the stability of the nonzero fixed point in Equation (D6). The nonzero fixed point becomes stable when the $\frac{d\langle \dot{r}\rangle }{d\langle r\rangle }{|}_{r^{*}}<0$. Hence, if the condition

(D8) δ(2γ−1)<α

is satisfied, REPIN populations can persist at high *hgt* rates (Figure D1).

From Equation (D7), the bacterial population size decreases as the result of carrying REPINs for

(D9) $$1-xy-\frac{(y+1)x}{2}\ln{\left(xy\right)}^{-1}<0,$$

where x=δ/α and y=2γ−1. Surprisingly, there is a finite range in which REPINs persist even though they reduce the bacterial population size, satisfying both conditions Equations (D8) and (D9).

Note that for α≪h, the steady-state distribution again follows Equation (C9), and thus if λ≫h, a nonzero REPIN solution cannot be achieved. It is because too high randomness makes the system lose all REPINs before any recovery mechanism (growth or mixing from *hgt*). Hence, for observing nontrivial solutions, we need higher *α* (inducing faster growth of bacteria carrying REPINs) or higher *hgt* rates (faster mixing).

#### Low *hgt* regime

Here, we investigate whether the expected REPIN number obtained in Equation (D6) is a good approximation for REPIN numbers when *hgt* rates are low. We focused on the most extreme case of *h *=* *0. First, we randomly draw three free parameters (benefit effect *w*, fatality *γ*, and benefit strength *α*) that lead to observable REPIN numbers (91≤〈r〉≤323). For 500 randomly selected parameter sets, we numerically get the distribution *b_r_* at $t=10^{6}$ with an initial condition $b_{r}(0)=\delta_{r,100}$. After obtaining numerical results, we compared the REPIN numbers obtained for *h *=* *0 with values calculated for high *hgt* rates. Because REPIN numbers without *hgt* are always smaller than for high *hgt* rates, we express the difference between the REPIN numbers as a proportion (Figure D2). 100% indicates the obtained REPIN numbers without *hgt* are the same as for high *hgt*, meaning the expression in Equation (D6) is a good estimation. 0% means REPINs go extinct without *hgt*. When the bacterial population size exceeds the carrying capacity (carrying a REPIN population is beneficial), the REPIN population size for high *hgt* rates and no *hgt* are similar (Figures D2 and D3).

#### Concerning the value of *λ*

The above analysis shows that our qualitative results should not depend on the value of *λ*. At least as long as *λ* is lower than the upper bound of our estimate for *λ* (*i.e.*, $6.38\times 10^{-8}$). This is because, even if *h* were to be lower than *λ* (we have assumed that $h=10^{-6}$ in the last figures of the manuscript), then *δ* as well as *α* would still be in the range of $10^{-8}$, which is also in the range of *λ*. As long as *λ* is in the range of *α* or *δ* the qualitative results of our model should remain unchanged.

### E Integrating RAYT dynamics

Different number of RAYTs may induce the different transposition rates; the higher number of RAYTs will increase the duplication chance. To include such transposition rate dynamics, we integrate the RAYTs in our model. Now each bacterium can be distinguished by the number of REPINs and RAYTs, *r* and *l*. We describe the dynamics of subpopulations expressed as $b_{r,l}$, the relative number of bacteria carrying *r* REPINs and *l* RAYTs.

We assume that the number of RAYTs does not affect any rates but the transposition rate. Using *δ* in the original model as a unit of transposition rate and assumed that the transposition rate with *l* RAYTs linearly increases with *l*, $\delta_{l}=\delta \cdot l$. As like REPIN amplification and deletion, RAYT also can be amplified or be deleted with rate *ϵ*. Again, if RAYT loses its all copy numbers, the amplification of RAYT cannot happen. For the sake of the simplicity, we consider maximally two RAYTs in a bacterium; each bacterium has either 0, 1, or 2 RAYTs in it. Since RAYTs only affect the transposition rate, the extend model dynamics is similar to the original model. However, transposition rates depending on RAYT numbers and transitions between RAYT numbers appear.

To get the full dynamics of the integrated model, we will focus on the bacterial dynamics without RAYT amplification and deletion first. Then, at a given number of RAYTs *l*, we can write the dynamics as

(E1) $$\begin{array}{c} {\dot{b}}_{r,l}=g(r,l)b_{r,l}+\delta l(1-\gamma )[(r-1)b_{r-1,l}-rb_{r,l}]\\ +\lambda [(r-1)b_{r-1,l}+(r+1)b_{r+1,l}-2rb_{r,l}]\\ +h[(r+1)b_{r+1,l}-rb_{r,l}]+h\langle r\rangle [b_{r-1,l}-b_{r,l}]\\ \equiv F(r,l). \end{array}$$

Here, the growth function g=g(r,l) and the average REPIN numbers 〈r〉 can be calculated in the similar ways with the original model, g(r,l)=(1−B−rγδl+Γ(r)) and $\langle r\rangle =\sum_{r,l}rf_{r,l}$ where $f_{r,l}=b_{r,l}/\sum_{r,l},b_{r,l}$. In Equation (E1), the first term comes from the growth rate and the second one describes the flow from the successful duplication event. As you can see, now the transposition rate is written as $\delta_{l}=\delta \cdot l$. Now we turn on the RAYT amplification and deletion. Setting the same amplification and deletion rates *ϵ*, we can get the full dynamics of $b_{r,l}$:

(E2) $${\dot{b}}_{r,l}=F(r,l)+\epsilon [\delta_{l,2}b_{r,l-1}+(1-\delta_{l,2})b_{r,l+1}-\delta_{l,1}b_{r,l}-(1-\delta_{l,0})b_{r,l}],$$

where $\delta_{n,m}$ is the kronecker delta,

(E3) $$\delta_{n,m}=\{\begin{array}{c} 1\;\text{for}\;n=m\\ 0\;\text{for}\;n\neq m. \end{array}$$

The first term in Equation (E2) is the dynamics without amplification and deletion and the other terms are added due to the changes of RAYT numbers. For all possible l∈{0,1,2}, we can write

(E4) $${\dot{b}}_{r,l}=\{\begin{array}{cc} F(r,0)+\epsilon b_{r,1}\; & \text{for}\;l=0,\\ F(r,1)+\epsilon [b_{r,2}-2b_{r,1}]\; & \text{for}\;l=1,\\ F(r,2)+\epsilon [b_{r,1}-b_{r,2}]\; & \text{for}\;l=2. \end{array}$$

This extended model can capture the changes of transposition rate. A full analysis of the extended system is beyond the current scope of this study. We numerically checked the existence of nonzero REPINs for a chosen parameter set, see Figure E1.
